# P-1259. Voriconazole Dosing and Therapeutic Drug Monitoring (TDM) in Pre and Post Urgent Orthotopic Liver Transplant Recipients with Decompensated Cirrhosis

**DOI:** 10.1093/ofid/ofaf695.1450

**Published:** 2026-01-11

**Authors:** Wesley J Hoffmann, Shemual Tsai, William L Musick, Luma Succar, Masayuki Nigo

**Affiliations:** Houston Methodist Hospital, Houston, TX; Houston Methodist Hospital, Houston, TX; Houston Methodist Hospital, Houston, TX; Houston Methodist Hospital, Houston, TX; Houston Methodist Hospital, Houston, TX

## Abstract

**Background:**

Voriconazole is a triazole antifungal metabolized by the cytochrome P450 system in the liver. For mild to moderate liver impairment, the manufacturer recommends reduction in the maintenance dose, but no dose recommendations are provided for patients with severe liver impairment. Previous literature showed that a significant dose reduction is needed in the setting of severe liver failure; however, there is limited data on empiric dosing in patients undergoing orthotopic liver transplant (OLT) evaluation and surgery.

**Table 1:**
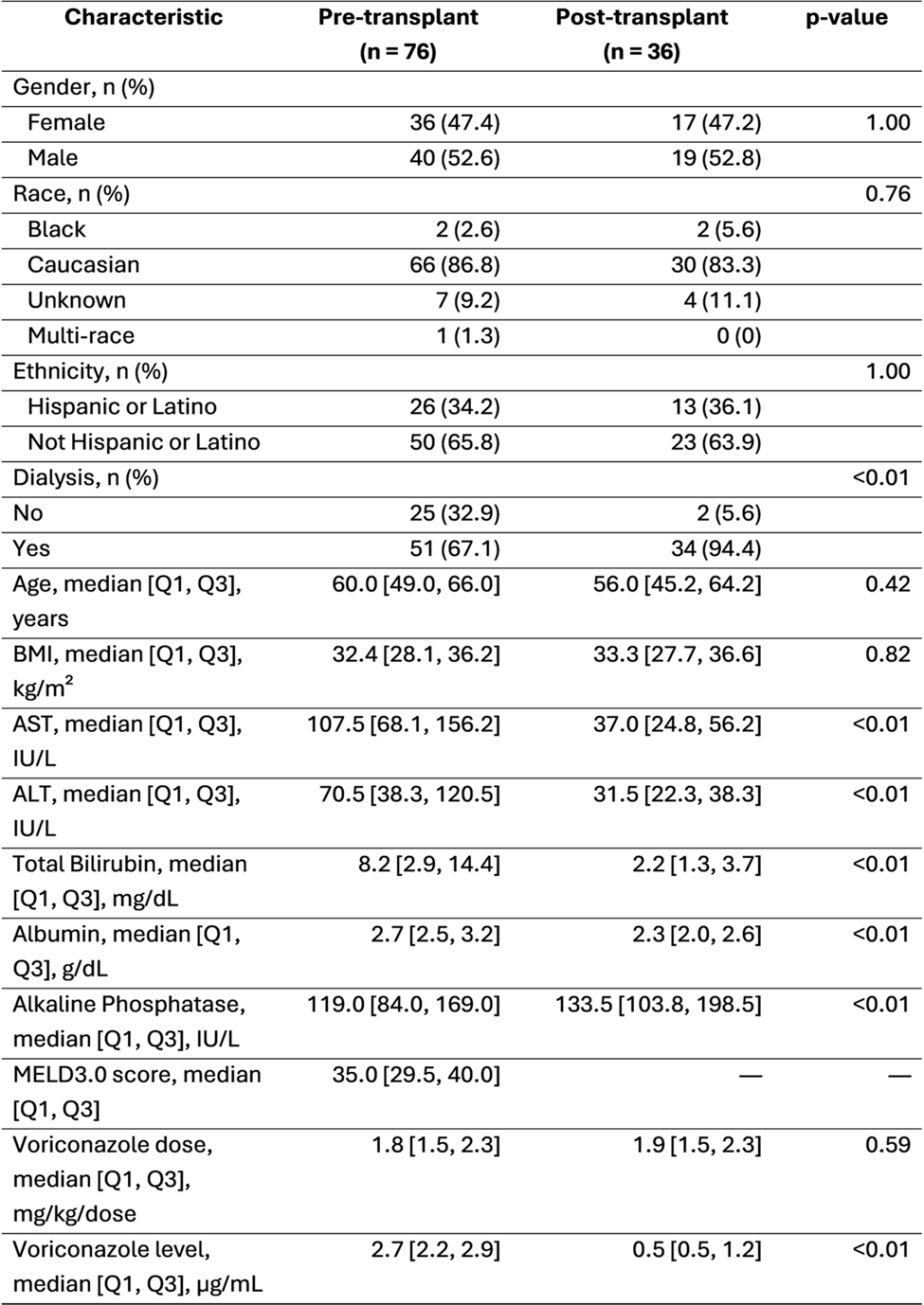
Patient Characteristics Based on Pre and Post Liver Transplant Abbreviations: BMI, body mass index; AST, aspartate aminotransferase; ALT, alanine aminotransferase; MELD, Model for End-stage Liver Disease; Q1, first quartile; Q3, third quartile. P-values were calculated using the Chi-square or Fisher’s exact test for categorical variables and the Wilcoxon rank-sum test for continuous variables.

Each patient was included only once per group. For patients with multiple measurements, only the first value was used for this analysis. Some patients appear in both the pre- and post-transplant groups if they underwent liver transplantation.

**Figure 1:**
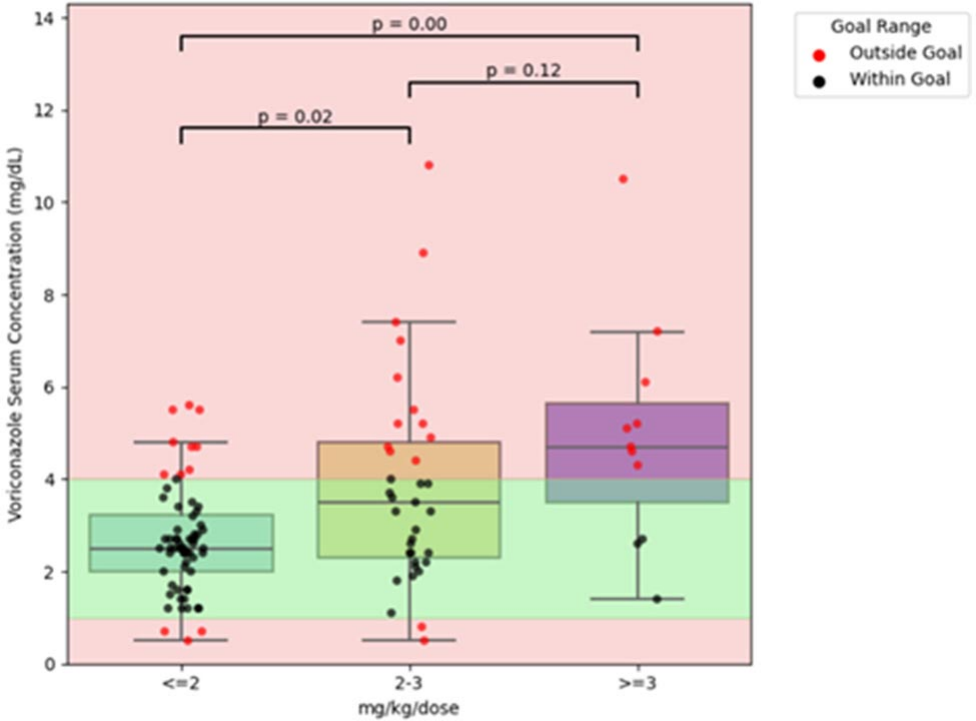
Pre-liver transplant voriconazole serum concentrations based on the dosage

**Methods:**

This is a retrospective study to assess voriconazole dosing and therapeutic drug monitoring (TDM) in patients with advanced cirrhosis who underwent urgent OLT evaluation and/or surgery and were receiving voriconazole for invasive fungal infection prophylaxis from 11/2023 to 11/2024. Patients were included if voriconazole trough levels were collected at least 7 days after therapy initiation pre-operatively, and at least 5 days after OLT. Levels were considered subtherapeutic and supratherapeutic when the level was < 1 mcg/mL or >4 mcg/mL, respectively.

**Figure 2:**
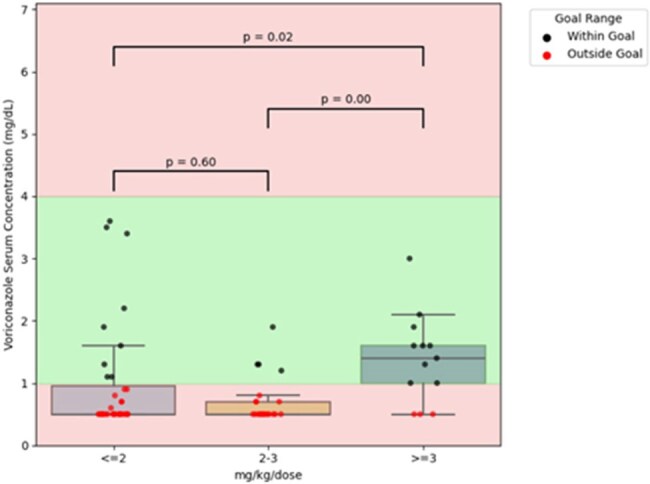
Post-liver transplant voriconazole serum concentrations based on the dosage

**Results:**

A total of 81 patients were identified in the study period; pre-OLT: 76 patients with 124 voriconazole levels and post-OLT: 36 patients with 71 levels. Table 1 summarizes the patient characteristics in both groups. The pre-OLT group had more abnormal liver enzyme results, as expected, with a high median MELD score of 35 (IQR 29 – 40). Figure 1 shows voriconazole trough levels based on different weight-based dosing regimens. In the pre-OLT setting, doses less than 2 mg/kg/dose were more likely associated with therapeutic voriconazole levels. Supratherapeutic levels were observed in 41 patients (avg. dose of 2.4 mg/kg/dose), of which five patients required therapy interruption. In the post-OLT group, only 23 of 71 levels fell within the therapeutic range, with an average dose of 2.8 mg/kg/dose (Figure 2).

**Conclusion:**

We found that empiric voriconazole dosing in patients with advanced cirrhosis should not exceed 2 mg/kg/dose, to avoid risk of accumulation and potential toxicity. In post-OLT patients, it may be reasonable to revert to standard manufacturer-recommended dosing. TDM is essential in managing these patients effectively.

**Disclosures:**

All Authors: No reported disclosures

